# 硼酸官能化金属-有机骨架磁性纳米复合材料的制备及其在茶叶农药残留检测中的应用

**DOI:** 10.3724/SP.J.1123.2021.06003

**Published:** 2021-10-08

**Authors:** Fengya WANG, Liang FENG

**Affiliations:** 1.中国科学院大连化学物理研究所, 中国科学院分离分析化学重点实验室, 辽宁 大连 116023; 1. CAS Key Laboratory of Separation Science for Analytical Chemistry, Dalian Institute of Chemical Physics, Chinese Academy of Sciences, Dalian 116023, China; 2.中国科学院大学, 北京 100049; 2. University of Chinese Academy of Sciences, Beijing 100049, China

**Keywords:** 磁性纳米粒子, 硼亲和, 金属有机骨架, 气相色谱-质谱, 农药残留, 茶叶, magnetic nanoparticles, boron affinity, metal organic framework (MOF), gas chromatography-mass spectrometry (GC-MS), pesticide residues, tea

## Abstract

在茶叶的农药残留检测中,茶多酚及色素具有很强的基质效应,严重影响了色谱检测结果。该文将Fe_3_O_4_磁性纳米粒子与硼酸官能化金属有机骨架(BA-MOF)材料相结合,制备出一种对茶多酚等基质具有高效捕获能力的吸附材料Fe_3_O_4_@BA-MOF。结合气相色谱-质谱联用技术,建立了一种茶叶样品中农药残留的有效分析方法。通过在金属有机骨架结构中引入硼酸配体,将其作为顺式二醇的识别位点,实现对茶多酚的高效捕获。这种新型材料具有快速磁分离,比表面积高,功能位点丰富等优点。通过傅里叶变换红外光谱、扫描电子显微镜以及X射线粉末衍射仪对制备材料进行了表征。同时对吸附剂的固相吸附条件(溶液pH、吸附剂用量、吸附时间)进行了优化,结果显示,50 mg Fe_3_O_4_@BA-MOF吸附材料可在5 min内去除74.58%茶多酚,溶液pH在7.0时效果最佳。利用硼亲和与茶多酚之间的可逆化学反应,通过调节溶液pH,可使Fe_3_O_4_@BA-MOF具有循环利用性,经过4次循环使用后仍具有优异的吸附性能。引入Fe_3_O_4_磁性纳米粒子,使其在样品前处理过程中表现出快速磁响应特性,提高前处理效率。在茶叶农药残留检测的实际应用中,经过Fe_3_O_4_@BA-MOF前处理后,10种农药的平均加标回收率为75.8%~138.6%, RSD为0.5%~18.7%(*n*=3)。研究结果表明,所制备的Fe_3_O_4_@BA-MOF纳米复合材料可以特异性吸附茶多酚基质,在茶叶的农药残留检测中具有净化基质,提高检测效率的功能,适用于茶叶中农药的检测分析。

## Abstract (en)

Tea is one of the most popular beverages worldwide, and its quality is often affected by the excessive pesticide residues during production. During the detection of pesticide residues in tea by chromatography-mass spectrometry and other methods, a strong matrix effect attributed to tea polyphenols and pigments is observed, which seriously impacts the analysis results. In this study, Fe_3_O_4_ magnetic nanoparticles and boric acid-functionalized metal organic framework (BA-MOF) materials were combined to prepare a highly efficient adsorbent Fe_3_O_4_@BA-MOF for capturing tea polyphenols and pigments. An effective analysis method for pesticide residues in tea samples in combination with gas chromatography-mass spectrometry was established. The introduction of boronic acid ligands into the metal organic framework, as the recognition site of *cis*-diols, enhanced the polyphenol capture ability. Adsorption of the pigment in the matrix was achieved through *π-π* interactions between the MOF ligand and the pigment. This new material has significant advantages such as rapid magnetic separation, large surface area, and abundant functional sites.

Fe_3_O_4_@BA-MOF was prepared by employing simple conditions and characterized by Fouriertransform infrared spectroscopy, scanning electron microscopy, and X-ray diffractometry to identify its functional groups and morphology. After investigating the adsorption effect of different doses of Fe_3_O_4_@BA-MOF adsorbents (5, 10, 30, 50, and 80 mg) on tea polyphenols, 50 mg of the adsorbent was added to the tea matrix and shaken thoroughly. The tea polyphenol content in the matrix solution was determined using an ultraviolet spectrophotometer. The polyphenols were reduced by 74.58% within 5 min. The effect of solution pH (2.0, 4.0, 6.0, and 7.0) on the adsorption efficiency was investigated, and pH 7.0 was chosen as the optimal condition. By adjusting the pH of the solution, Fe_3_O_4_@BA-MOF could be recycled, and it maintained the excellent adsorption performance after four cycles of use. The introduction of Fe_3_O_4_ magnetic nanoparticles led to rapid magnetic response characteristics during sample pretreatment and improved the pretreatment efficiency. In the actual application of tea pesticide detection, after Fe_3_O_4_@BA-MOF pretreatment, the average recovery rates of the ten pesticides were in the range of 75.8%-138.6%, and the RSD was in the range of 0.5%-18.7% (*n*=3).

The Fe_3_O_4_@BA-MOF nanocomposite prepared by introducing the boric acid ligand into the MOF structure and incorporating Fe_3_O_4_ magnetic nanoparticles could specifically adsorb the tea polyphenol matrix. When applied to the detection of pesticide residues in tea, it purifies the matrix and improves the detection efficiency, thus being suitable for the detection and analysis of pesticides in tea.

茶叶具有降低血压、预防糖尿病、骨质疏松症和心血管疾病的功效^[1]^。然而在茶叶生产过程中,为了防治害虫、杂草和病害,不可避免地需要使用杀虫剂。过量杀虫剂的喷洒会损害人体健康,影响茶叶的质量,带来产品销售和出口的损失^[2]^。因此,茶叶中农药残留量的测定对于公共卫生安全具有十分重要的意义。许多国家和国际组织已经颁布了法定的茶叶农药最大残留量(MRL),以保护消费者的健康并规范国际茶叶贸易^[3]^。在使用气相色谱或液相色谱法对茶叶中农药残留进行检测时,茶多酚等大量基质成分会对检测结果造成严重干扰,导致假阴性或假阳性,甚至也会对仪器离子源造成污染^[4,5]^。

复杂基质中痕量目标成分的检测主要依赖于样品前处理对基质成分进行吸附净化。目前常用的样品前处理方法主要包括固相萃取(SPE)^[6]^、凝胶渗透色谱法(GPC)^[7]^、液-液微萃取^[8]^、超临界流体萃取(SFE)^[9]^等。在这些方法中,基于分散固相萃取(d-SPE)的QuEChERS方法^[10]^已广泛用于复杂样品前处理。在该技术中,关键组成是吸附净化剂。其中,经典的吸附剂包括乙二胺-*N*-丙基硅烷(PSA),石墨炭黑(GCB)和十八烷基(C_18_)。然而对于不同类型的样品基质,通常需要组合多种吸附剂,没有吸附专一性。因此,根据茶叶中主要的基质成分,开发针对茶叶基质的特异性吸附材料,在提高净化效率方面具有重要意义。

金属有机骨架(MOF)作为近年来新兴的吸附材料,具有比表面积大,孔径可调谐等性质,在分离领域显示出了广阔的前景^[11]^,对色素、染料等化合物展现出优异的吸附性能^[12]^。硼亲和材料(BA)是一种可以选择性分离富集顺式二羟基生物分子的功能性材料,硼酸配体可以可逆地与顺式二羟基结构化合物(如糖蛋白、糖类、核苷酸等活性成分)相结合,在食品检测、生物分离等方面应用广泛。茶多酚含有大量顺式二羟基结构^[13]^,可通过硼亲和材料选择性地分离富集^[14]^。开发具有硼酸亲和基团的MOF材料,对于茶多酚等分子的吸附分离具有重要的意义。近年来,通过引入配体片段(5-硼苯-1,3-二羧酸(BBDC))方式协同参与功能性MOF的合成,引起了较大的关注。Gu等^[15]^采用一种简便的金属-配体-片段共组装方法来制备具有硼酸官能化的Cr(Ⅲ)基MOF,所得的MOF纳米颗粒在分离顺式二醇结构的生物分子方面展现出优异的效果,为功能化MOF的制备提供了一种简便而有效的方法。但是,由于MOF颗粒尺寸较小,在固液分离或过滤过程中处理困难,且易于泄露。此外,MOF材料中使用重金属(例如Cr(Ⅲ))具有高毒性,对环境造成潜在污染^[16]^,因此,使用低毒的过渡金属(例如Zn(Ⅱ)),采用引入配体片段的策略制备功能性MOF吸附材料,在复杂样品的前处理分离中将具有重要的应用价值。

在这项研究中,我们将Fe_3_O_4_磁性纳米粒子与具有高吸附性能的锌基MOF结构相结合,同时引入硼酸配体,成功构建硼酸官能化金属-有机骨架磁性纳米复合材料(Fe_3_O_4_@BA-MOF),将其应用于茶叶农药残留检测过程中茶多酚等基质的特异性净化吸附。对复合材料吸附条件进行优化后,结合气相色谱-质谱联用技术,建立了一种茶叶样品中农药残留的有效分析方法。

## 1 实验部分

### 1.1 仪器、试剂与材料

Persee TU-1901紫外分光光度计(北京普析通用仪器有限责任公司); JEM-7800F扫描电子显微镜(JEOL,日本); LC-20A型HPLC系统(Shimadzu,日本); 8890A-5977B气相色谱-质谱联用仪(Agilent,美国); FTIR-Nicolet iS50傅里叶变换红外光谱仪(Thermo fisher,美国); X射线粉末衍射仪(PANalytical,德国)。

六水合氯化铁、乙酸钠、己二醇、聚乙二醇、*N*,*N*'-二甲基甲酰胺(DMF)、对苯二甲酸、5-硼苯-1,3-二羧(分析纯,上海阿拉丁生化科技股份有限公司);弗罗里硅土(Florisil)、乙二胺-*N*-丙基硅烷(PSA)(上海麦克林生化科技有限公司);纯净水(杭州娃哈哈集团有限公司);乙腈、甲醇(色谱纯,国药集团化学试剂有限公司);农药混合标准溶液(包括4-溴-3,5-二甲苯基-*N*-甲基氨基甲酸酯-1(4-bromo-3,5-dimethylphenyl-*N*-methylcarbamate-1)、三正丁基磷酸盐(tri-iso-butyl phosphate)、蔬果磷(dioxabenzofos)、脱乙基另丁津(desethyl-sebuthylazine)、合成麝香(musk ambrette)、麦穗灵(fuberidazole)、2-甲-4-氯丁氧乙基酯(2-methyl-4-chlorobutoxyethyl ester)、灭菌磷(ditalimfos)、威菌磷(triamiphos)及苄呋菊酯(resmethrin),纯度99%,上海安谱实验科技股份有限公司);绿茶(大连市家乐福超市)。

### 1.2 Fe_3_O_4_@BA-MOF纳米复合材料的制备

1.2.1 Fe_3_O_4_磁性纳米粒子的制备

根据文献^[17]^,采用经典水热合成法制备Fe_3_O_4_磁性纳米粒子。首先将六水合氯化铁(1.35 g)溶解在40 mL己二醇中,形成澄清的橙黄色溶液;然后向上述溶液中添加3.6 g无水乙酸钠和1.0 g聚乙二醇,将混合物剧烈搅拌30 min,并密封在反应釜中,在200 ℃下反应10 h,得到Fe_3_O_4_磁性纳米粒子。通过磁铁收集反应得到的Fe_3_O_4_磁性纳米粒子,分别使用超纯水及乙醇反复洗涤样品后,置于真空干燥箱中进行真空干燥。

1.2.2 Fe_3_O_4_@BA-MOF纳米复合材料的制备

根据文献^[18]^,将0.05 g Fe_3_O_4_与3.0 g六水合硝酸锌混合于15 mL DMF试剂中,常温搅拌4 h。随后将配体对苯二甲酸(45 mg)及5-硼酸-1,3-二羧酸(4.5 mg)加入体系中。将混合物搅拌30 min后加入反应釜中,密封,120 ℃水热反应6 h。使用磁铁收集反应得到的Fe_3_O_4_@BA-MOF纳米复合材料,分别使用超纯水及乙醇反复洗涤样品后,置于真空干燥箱中进行真空干燥。

### 1.3 样品前处理

将茶叶样品研磨成粉末,混匀后称取10 g加入50 mL离心管中,加入30 mL去离子水(60 ℃)超声提取30 min。然后将混合物以4000 r/min离心20 min。收集上清液,作为茶叶提取液,用于后续净化处理。

### 1.4 茶叶中基质的磁性吸附及净化

称取50 mg的Fe_3_O_4_@BA-MOF,加入2 mL茶叶提取液,调节pH至7.0。振荡10 min后,通过外部磁铁将吸附材料吸引至管壁,吸取管中澄清液体。加入0.5 mL乙腈提取溶液中的农药,振荡30 s后加入无水硫酸镁(500 mg), 3000 r/min离心后取有机相进行气相色谱-质谱联用分析。

### 1.5 茶多酚的检测条件

根据文献^[19]^,使用分光光度计在273 nm处对茶叶中的茶多酚总量进行检测,并使用高效液相色谱仪对茶多酚吸附情况进行表征。根据文献^[20]^分析方法,使用SPD-M20A光电二极管阵列检测器,分析柱为C_18_柱(250 mm×4.6 mm, 5 μm, Waters,美国)。分析条件如下:流动相由甲醇-水(20;80, v/v)组成;流速为1 mL/min;柱温保持在25 ℃;检测波长设置为273 nm;进样量为10 μL。

### 1.6 农药的气相色谱-质谱检测条件

色谱柱:HP-5(30 m×0.25 mm×0.25 μm, Agilent,美国)石英毛细管柱。色谱柱温度程序:40 ℃保持1 min,以30 ℃/min升温至130 ℃,然后以5 ℃/min升温至250 ℃,再以10 ℃/min升温至300 ℃,保持5 min;进样口温度:290 ℃;进样量:1 μL;电子轰击源:70 eV;离子源温度:230 ℃;传输线温度:280 ℃;采用选择离子监测模式(SIM)扫描。

## 2 结果与讨论

### 2.1 Fe_3_O_4_@BA-MOF纳米复合材料的制备

MOF由于其较大的比表面积,孔径可调节性和高孔隙率而被广泛应用到分析检测过程中样品的分离富集。通过配体与目标物之间的*π-π*相互作用力,可快速吸附色素,染料等化合物^[21]^。在此基础上,将具有茶多酚特殊识别功能的硼酸基团(5-硼苯-1,3-二羧酸)引入MOF材料的有机配体中,可有效提高MOF的识别选择性^[15]^,应用于茶叶农药残留分析过程中的基质净化吸附。

对制备的Fe_3_O_4_及Fe_3_O_4_@BA-MOF样品进行扫描电镜分析,观察其形貌。如图1a所示,通过水热法制得的Fe_3_O_4_粒子分布均匀,粒径分布范围在400~500 nm之间;经过以硼酸为配体的锌基MOF材料修饰后(见图1b),可以清楚看到Fe_3_O_4_磁性粒子均匀分布于MOF内部。

**图1:**
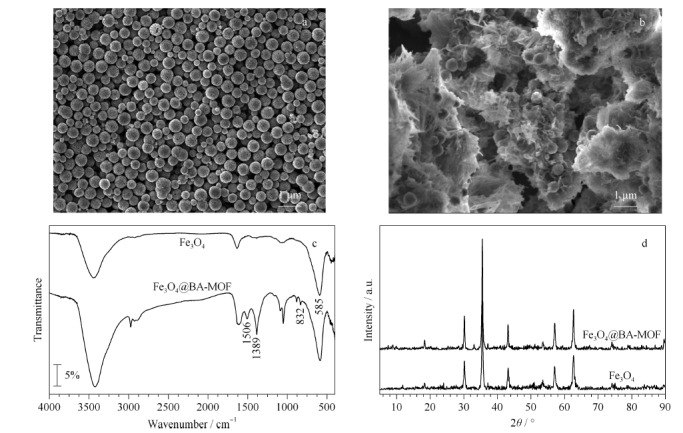
Fe_3_O_4_和Fe_3_O_4_@BA-MOF的表征结果

通过FT-IR对制备的Fe_3_O_4_及Fe_3_O_4_@BA-MOF复合材料的基团构成进行表征。如图1c所示,585 cm^-1^处的吸收峰可归因于Fe_3_O_4_中Fe-O-Fe的伸缩振动^[22]^,这一特征峰在Fe_3_O_4_@BA-MOF复合材料中仍可发现。在Fe_3_O_4_@BA-MOF的红外谱图中可清楚地观察到1506 cm^-1^及832 cm^-1^处MOF配体对苯二甲酸的-C=O与苯环伸缩振动峰,同时在1389 cm^-1^处存在硼酸配体中的B-O键的伸缩振动峰^[23]^。

另外,使用XRD对制备的Fe_3_O_4_及Fe_3_O_4_@BA-MOF纳米复合材料的晶体结构进行了表征。如图1d所示,Fe_3_O_4_在2*θ*=30.2°、35.6°、43.3°、53.7°、57.2°及62.8°处出现了特征峰,分别代表(220)、(311)、(400)、(422)、(511)及(440)晶面。与Fe_3_O_4_相比,Fe_3_O_4_@BA-MOF在10°附近出现了特征峰,这是由BA-MOF形成引起的,与文献^[22]^报道一致。

### 2.2 Fe_3_O_4_@BA-MOF吸附性能

如图2a所示,将Fe_3_O_4_@BA-MOF吸附材料加入茶叶提取液后,溶液中茶多酚含量迅速降低,在5 min内减少74.58%。随着吸附时间的延长,逐渐达到平衡,在20 min内Fe_3_O_4_@BA-MOF对茶多酚的吸附效果达到78.78%。

**图2:**
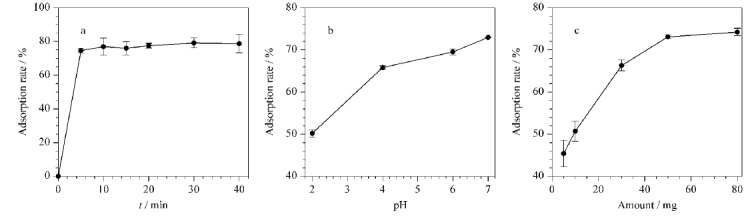
(a)吸附时间、(b)溶液pH、(c)吸附剂添加量对茶多酚的吸附效率影响(*n*=3)

溶液pH值可能影响Fe_3_O_4_@BA-MOF中硼酸配体对茶多酚的亲和力,是影响茶叶基质吸附效果的关键因素。因此,首先考察不同pH值(2.0、4.0、6.0、7.0)条件下,溶液中茶多酚的吸附效率。如图2b所示,在低pH值的酸性条件下,Fe_3_O_4_@BA-MOF对茶叶基质的吸附效率仅为50%。随着溶液pH增大,材料对茶多酚的吸附效果逐渐增强,这一现象与文献中的报道一致^[22]^。由于部分农药在碱性条件下会发生分解,因此将提取条件定为pH 7.0。

在这项研究中,锌基MOF材料为基质吸附过程提供了较大的比表面积。然而样品制备过程中,使用过量的吸附材料可能对目标农药的回收率和共提取物的净化效率产生直接影响^[24]^。因此,在确定吸附pH值后,本实验对吸附剂的用量也进行了考察。如图2c所示,在2 mL的茶叶提取液中,随着吸附剂用量逐渐增加(5、10、30、50、80 mg), Fe_3_O_4_@BA-MOF对茶多酚的吸附效率也逐渐增强。当吸附剂用量高于50 mg时,吸附效果变化不明显。

综上,确定Fe_3_O_4_@BA-MOF的吸附条件为吸附时间5 min,溶液pH为7.0,吸附剂添加量为50 mg,并在此条件下进行后续实验。

### 2.3 重复使用性

作为色谱分离中的经典材料,硼酸亲和材料可通过溶液pH值,控制硼酸配体与顺式二醇化合物之间可逆的共价相互作用,实现目标物的“捕获和释放”过程^[25]^。在碱性条件下选择性捕获顺式二醇化合物,形成硼酸酯结构络合物,而在酸性条件下自动解离^[26]^。利用硼酸亲和材料的pH响应性,我们通过使用0.1 mol/L NaOH及HCl调节溶液pH,在pH 6.0的条件下对Fe_3_O_4_@BA-MOF中吸附的茶多酚进行解吸。通过4个连续的吸附再生循环对Fe_3_O_4_@BA-MOF进行评估,与初始值相比,经过4个循环后,Fe_3_O_4_@BA-MOF对茶多酚的吸附效率仅降低了2.57%,少量的损失可能归因于循环过程中特异性结合位点的减少以及基质中色素在MOF晶体结构中的孔隙填充^[27]^。总的来说,Fe_3_O_4_@BA-MOF在4个循环中具有良好的再生能力。

### 2.4 与商品化吸附材料的效果对比

在茶叶的前处理方法中,Florisil及PSA作为经典的吸附材料,广泛应用于茶叶中色素、有机酸等基质的吸附处理^[24]^。实验考察了Fe_3_O_4_@BA-MOF与Florisil、PSA对茶多酚的吸附效果差异。称取相同质量的吸附材料,置于茶叶提取液中,充分振荡后,离心分离基质净化液,使用分光光度计测定溶液中茶多酚的含量。结果表明,Fe_3_O_4_@BA-MOF、Florisil及PSA对茶多酚的吸附效率分别是78.78%、57.97%及77.34%。作为正相吸附剂,弗罗里硅土在茶多酚的吸附方面缺乏特异性^[28]^,造成吸附效率较低。Fe_3_O_4_@BA-MOF与商品化PSA吸附剂对茶多酚去除效率相当,证明其在茶叶基质净化方面具有一定的实用性。

为避免污染质谱的离子源,采用高效液相色谱考察了净化效果,如图3所示,经过Fe_3_O_4_@BA-MOF净化后,色谱图中干扰成分的色谱峰显著减少,可有效地提高样品检测的准确度。同时,本方法中的样品前处理相较于传统固相萃取有着极大的简化。Fe_3_O_4_磁性纳米粒子的掺杂使吸附材料具有良好的顺磁性,在外部磁铁的作用下,快速分离吸附材料,提高前处理效率(见图3b, 3c)。

**图3:**
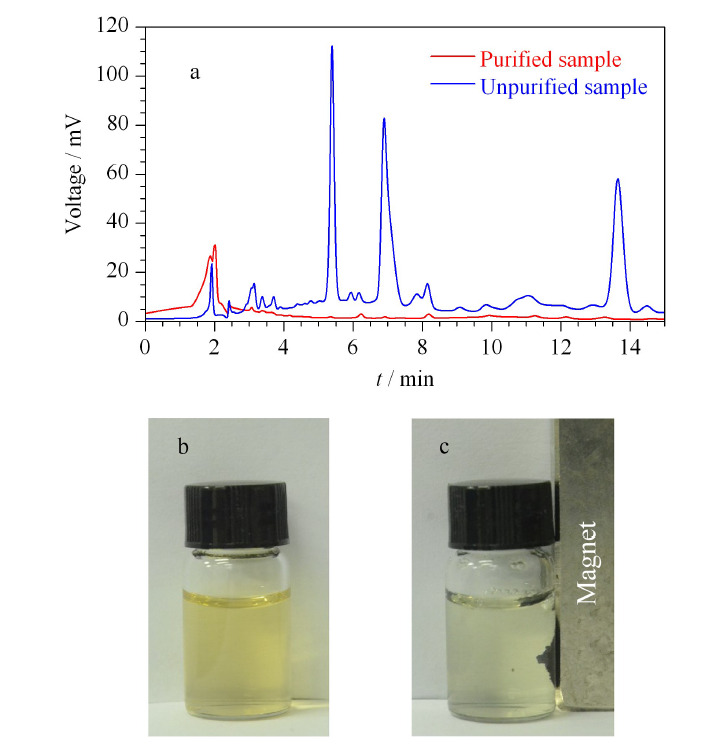
茶叶提取液经由Fe_3_O_4_@BA-MOF处理前后的 (a)高效液相色谱图及(b,c)磁分离照片

### 2.5 Fe_3_O_4_@BA-MOF在茶叶农残检测中的应用

在实际样品的气相色谱分析中,基质中的热敏性分析物可能会在仪器衬管和色谱柱的活性位点分解,导致峰形失真。同时,共提取物会与目标农药竞争进样口或柱头的金属离子、硅烷基等活性位点,导致色谱信号增强^[29]^。因此,研究开发针对样品基质的吸附材料,对实际应用中农药残留量的检测具有重要意义。取茶叶空白样品,经过前处理吸附净化后(依照1.3、1.4节方法)得到空白基质溶液。以空白基质溶液稀释农药混合标准溶液(根据文献^[30]^选择10种茶叶中常见的农药),配制成基质混合标准溶液,通过气相色谱-质谱联用仪进行检测。以目标物的峰面积(*y*)对质量浓度(*x*)进行线性回归分析,在1~10 mg/L的线性范围内,测得混合标准溶液中各农药的线性良好。以信噪比(*S/N*)=3确定10种农药的检出限(LOD),以*S/N*=10确定10种农药的定量限(LOQ)(见表1)。

**表1:** 10种农药的线性方程、质谱参数及检出限

Target	Retention time/ min	Quantitative ion (m/z)	Qualitative ions (m/z)	Linear equation	LOD/ (mg/L)	LOQ/ (mg/L)
BDMC-1	20.82	200	202, 201	y=2565.75x-1098.68	0.03	0.10
Tri-iso-butyl phosphate	10.25	155	139, 211	y=4212.41x-949.99	0.05	0.15
Dioxabenzofos	13.09	173	158, 145	y=7505.16x-2727.13	0.06	0.20
Desethyl-sebuthylazine	14.72	172	174, 186	y=14137.27x-2782.72	0.06	0.20
Musk ambrette	16.27	253	268, 223	y=5244.07x-2961.27	0.10	0.30
Fuberidazole	17.37	184	155, 129	y=11082.79x-2776.89	0.10	0.30
MCPA-butoxyethyl ester	20.82	300	200, 182	y=3405.81x-1459.83	0.03	0.10
Ditalimfos	21.86	130	148, 299	y=1678.82x+337.74	0.03	0.10
Triamiphos	24.81	160	294, 251	y=6048.10x-426.68	0.06	0.20
Resmethrin	26.41	171	143, 338	y=4967.23x-3187.67	0.06	0.20

*y*: peak area; *x*: mass concentration, mg/L.

在阴性茶叶样品中添加一定浓度的农药混合标准溶液,依照1.3、1.4节方法进行样品前处理,然后进行GC-MS分析。结果如表2所示,10种农药的平均加标回收率为75.8%~138.6%, RSD为0.5%~18.7% (*n*=3),表明该方法适用于茶叶中农药的检测分析。

**表2:** 10种农药加标回收率及精密度(*n*=3)

Target	Spiked/ μg	Found/ μg	Recovery/ %	RSD/ %
BDMC-1	3	2.82	94.3	16.3
	1	1.27	127.2	4.4
Tri-iso-butyl phosphate	3	2.82	93.9	12.6
	1	1.09	109.1	7.5
Dioxabenzofos	3	3.07	102.4	3.2
	1	0.92	91.2	0.6
Desethyl-sebuthylazine	3	2.48	82.8	6.2
	1	1.03	103.4	2.5
Musk ambrette	3	2.28	75.8	10.6
	1	1.27	126.7	0.5
Fuberidazole	3	2.28	75.7	6.4
	1	0.89	89.0	4.8
MCPA-butoxyethyl ester	3	2.82	94.0	12.8
	1	1.16	116.4	1.4
Ditalimfos	3	2.85	94.9	9.5
	1	0.88	88.7	8.9
Triamiphos	3	3.54	118.3	10.3
	1	1.32	131.6	10.0
Resmethrin	3	3.06	102.5	18.7
	1	1.38	138.6	0.7

## 3 结论

本研究针对茶叶农药残留检测中前处理复杂等问题,制备了一种特异吸附茶叶基质的磁性固相萃取吸附剂Fe_3_O_4_@BA-MOF。结合气相色谱-质谱,建立了一种有效测定茶叶中农药的方法。磁性纳米粒子与锌基MOF结构相结合,将硼酸配体引入MOF结构中,针对茶多酚等基质成分具有吸附特异性,制备方法简单,可以有效简化样品前处理步骤。本方法在针对茶叶中农药残留分析方面具有较高的应用价值和广阔的应用前景,但对于茶叶基质中咖啡碱、有机酸等成分消除能力较弱,仍需要进一步的探索研究。
